# OPPL-Galaxy, a Galaxy tool for enhancing ontology exploitation as part of bioinformatics workflows

**DOI:** 10.1186/2041-1480-4-2

**Published:** 2013-01-04

**Authors:** Mikel Egaña Aranguren, Jesualdo Tomás Fernández-Breis, Chris Mungall, Erick Antezana, Alejandro Rodríguez González, Mark D Wilkinson

**Affiliations:** 1 Ontology Engineering Group, School of Computer Science, Technical University of Madrid (UPM), Boadilla del Monte, 28660, Spain; 2 Biological Informatics Group, Centre for Plant Biotechnology and Genomics (CBGP), Technical University of Madrid (UPM), Pozuelo de Alarcón, 28223, Spain; 3 School of Computer Science, University of Murcia (UM), Murcia, 30100, Spain; 4 Genomics Division, Lawrence Berkeley National Laboratory, Berkeley, CA, 94720, US; 5 Department of Biology, Norwegian University of Science and Technology (NTNU), Høgskoleringen 5, Trondheim, N-7491, Norway

## Abstract

**Background:**

Biomedical ontologies are key elements for building up the Life Sciences Semantic Web. Reusing and building biomedical ontologies requires flexible and versatile tools to manipulate them efficiently, in particular for enriching their axiomatic content. The Ontology Pre Processor Language (OPPL) is an OWL-based language for automating the changes to be performed in an ontology. OPPL augments the ontologists’ toolbox by providing a more efficient, and less error-prone, mechanism for enriching a biomedical ontology than that obtained by a manual treatment.

**Results:**

We present OPPL-Galaxy, a wrapper for using OPPL within Galaxy. The functionality delivered by OPPL (*i.e.* automated ontology manipulation) can be combined with the tools and workflows devised within the Galaxy framework, resulting in an enhancement of OPPL. Use cases are provided in order to demonstrate OPPL-Galaxy’s capability for enriching, modifying and querying biomedical ontologies.

**Conclusions:**

Coupling OPPL-Galaxy with other bioinformatics tools of the Galaxy framework results in a system that is more than the sum of its parts. OPPL-Galaxy opens a new dimension of analyses and exploitation of biomedical ontologies, including automated reasoning, paving the way towards advanced biological data analyses.

## Background

Among the various steps that a typical life-sciences research cycle comprises, information extraction from raw data (and its dissemination to the community) remains as one of the most relevant ones. New biological insights are generated by combining information from different sources with the expertise of scientists. Nevertheless, integrating information and generating knowledge out of it is still a challenging task, as the information is frequently captured in computationally opaque formats and dispersed over the Web in resources with idiosyncratic schemas.

The Semantic Web [1] aims to overcome the issue of computationally opaque and disperse information in the Web with a set of technologies and standards defined by the W3C: RDF [2], SPARQL [3] and OWL [4]. Therefore, these standards are increasingly used by the Life Sciences community to integrate information (RDF), to query it (SPARQL), and to axiomatically encode consensus knowledge about such information in ontologies (OWL), in the so-called Life Sciences Semantic Web [5].

Biomedical ontologies are essential for the Life Sciences Semantic Web since they offer computationally processable and often Web-oriented representations of agreed-upon domain knowledge. The Gene Ontology (GO) [6] stands out as one of the most intensely curated and used biomedical ontologies; other important biomedical ontologies can be found at the Open Biological and Biomedical Ontologies Foundry [7], a project that hosts biomedical ontologies that follow certain design principles (reusability, orthogonality, *etc.*). Additionally, the National Center for Biomedical Ontology (NCBO) offers access to biomedical ontologies through BioPortal [8], including a set of Web Services.

Current biomedical ontologies support a broad range of tasks: axiomatically rich ontologies are used for intense automated reasoning [9], axiomatically lean ontologies act as vocabularies for Linked Data [10], and typically other functions in between [11]. In order to fulfill such functions, biomedical ontologies should be adapted to fit scientists’ requirements, especially when reusing pre-existing ontologies: addition or removal of axioms and entities, inference in relation to external ontologies, selective materialisation of inferred axioms, complex querying, and so forth.

Manipulating biomedical ontologies can be a laborious task since they are regularly growing in size [12] and axiomatic complexity [13]. Therefore, advanced tools are needed for efficiently performing such manipulation [14]. The Ontology Pre Processor Language (OPPL) [15] offers the possibility of automating this kind of ontology manipulation. By using OPPL, the ontologist can define the intended manipulation in an OPPL script as a series of additions or removals of axioms to be performed in a concrete ontology. Therefore, the use of OPPL makes the ontology manipulation process more efficient, sustainable and less error-prone.

OPPL capabilities have already been demonstrated: it has been used to build an ontology transformation service [16] and for applying [17-20] or detecting [21] Ontology Design Patterns (ODPs). Also, it is part of Populous, an application for adding content from spreadsheets to ontologies [22].

OPPL’s versatility and functionality cannot be exploited directly within the typical bioinformatics analyses. Galaxy, a Web server for combining various genomic-oriented tools into workflows [23], offers an ideal platform for making OPPL part of bioinformatics analyses. Therefore, we have developed OPPL-Galaxy, a tool to execute OPPL scripts from within Galaxy. OPPL-Galaxy enhances OPPL’s functionality, *i.e.* automated ontology manipulation, by providing the possibility of dynamically sending OPPL’s output, that is, an improved ontology, to other Galaxy tools (and making OPPL capable of consuming ontologies as input from other Galaxy tools).

This paper presents an overview of OPPL-Galaxy’s design and implementation, including tested use cases that provide a basis for creating more complex analyses. OPPL-Galaxy is also compared to other tools and its benefits and limitations are discussed.

## Implementation

### OPPL

OPPL implements its own syntax: an extension of the Manchester OWL Syntax (MOS) [24] that includes keywords like ADD (to add an axiom), REMOVE (to remove an axiom), SELECT (to select entities), and so on. An OPPL script defines a query and some actions that should be performed against the retrieved entities (see ‘Basic usage’ use case in Section). A query can combine variables (to be bound by a set of named entities) and actual named entities of the target ontology (OWL classes, properties, or individuals). An important constraint in OPPL specifies that every variable must resolve to a group of named entities (or none), not an anonymous OWL expression, to ensure that queries can be answered. The following types of queries can be defined in OPPL (all the queries mix variables with OWL expressions): 

• OWL queries that exploit automated reasoning.

• Syntactic OWL queries that only work with the asserted axioms.

• Queries that use a regular expression to match annotation values like rdfs:label.

**Figure 1:**
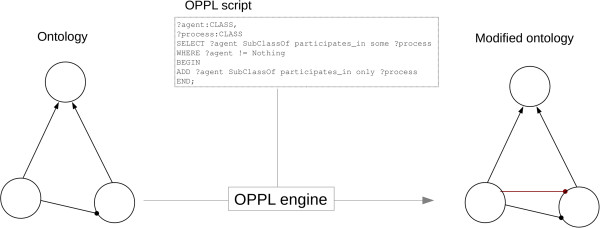
**Toy ontology for OWL rendering convention.** Toy ontology to illustrate the convention for representing abstract OWL structures in Figures depicting use cases. Above, the ontology is rendered using MOS; below, the ontology is rendered with the same convention as in Figures 2, 5, 6, 10 and 14. In those Figures, however, names of OWL entities are not included in the ontologies, since OPPL scripts act on absract structures (any axiomatic pattern that matches the query). Solid circle: named class; dotted circle: anonymous class; dot: named individual; solid arrow: subClassOf axiom; dotted arrow: triple (relation between individuals); line ending in circle: restriction (the small circle points to the filler class; there is no distinction between necessary and necessary/sufficient conditions)^a^.

**Figure 2:**
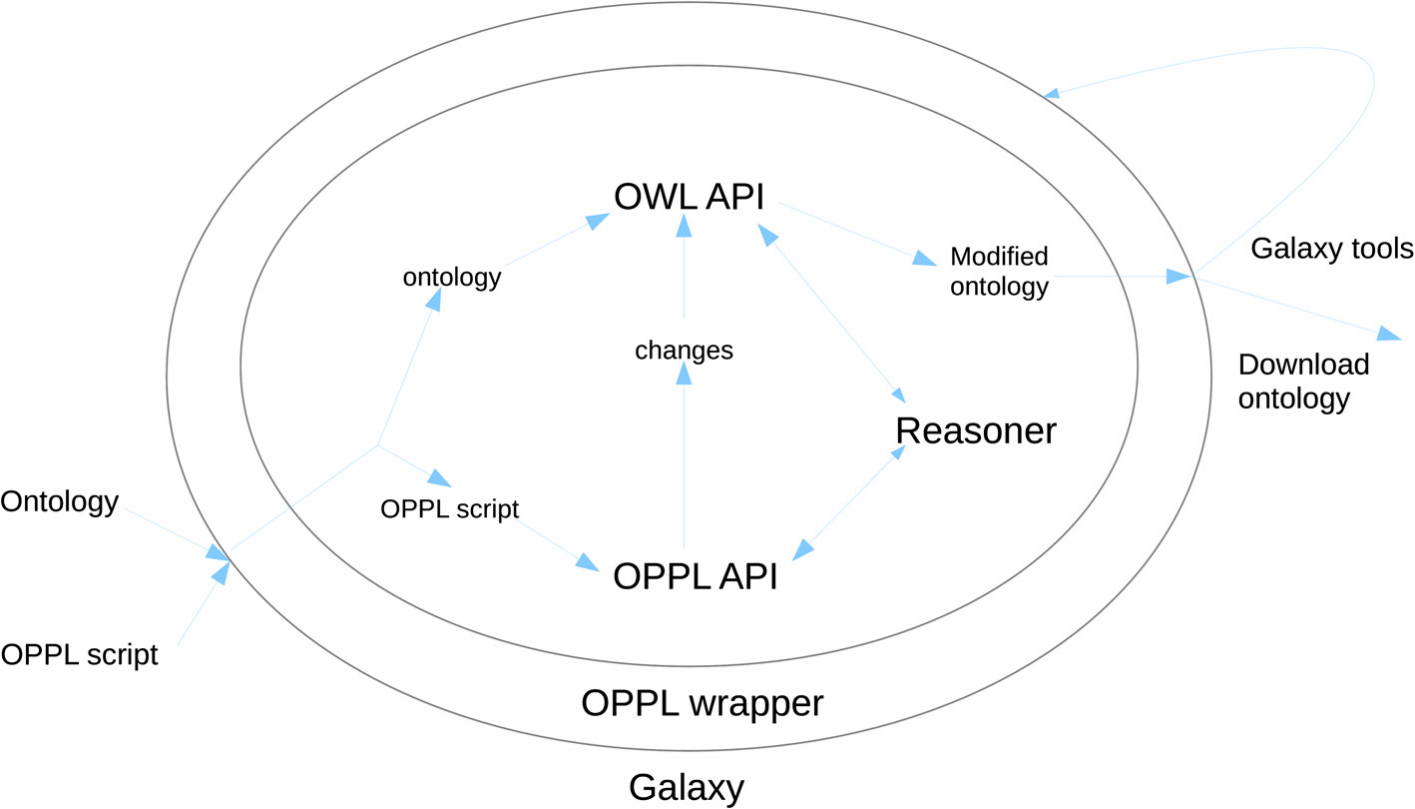
**OPPL pipeline.** The OPPL engine takes an ontology (circle group on the left) and an OPPL script (dotted square) as inputs, and performs the changes defined by the OPPL script on the input ontology, thereby generating a new output ontology (modified ontology, on the right).

The actions are based on the addition or removal of axioms of any complexity to/from entities retrieved by the query (OWL classes, properties, or instances). Once an OPPL script has been defined, the OPPL engine is passed this script and the ontology to be modified. The OPPL engine, in turn, modifies the ontology according to the changes defined in the OPPL script, generating a new ontology (Figures 1 and 2).

### Galaxy

Galaxy offers an open, Web-based platform for performing genomic analyses [23]. In Galaxy several tools can be combined, ranging from simple data manipulations (*e.g.* text manipulation) to complex analyses (*e.g.* statistical analysis of Next-Generation Sequencing data). Such a tool orchestration can be executed from within a single Web interface: the output of a tool can be sent to other tools as input, easing the construction of workflows by combining recurrent tasks. Moreover, a history of all performed actions is stored, so the analyses can be reproduced at any time and shared with other users. Galaxy workflows can be built from the users’ history and shared. Finally, the workflows can be migrated to other systems, like other Galaxy servers or myExperiment [25].

Apart from its functionality and ease of use, another appealing feature of Galaxy is its extensibility, allowing a straightforward integration of command-line tools: the only requirement is to create an XML file containing a description of the tool’s Web interface and inputs/outputs [26].

### OPPL-Galaxy

**Figure 3:**
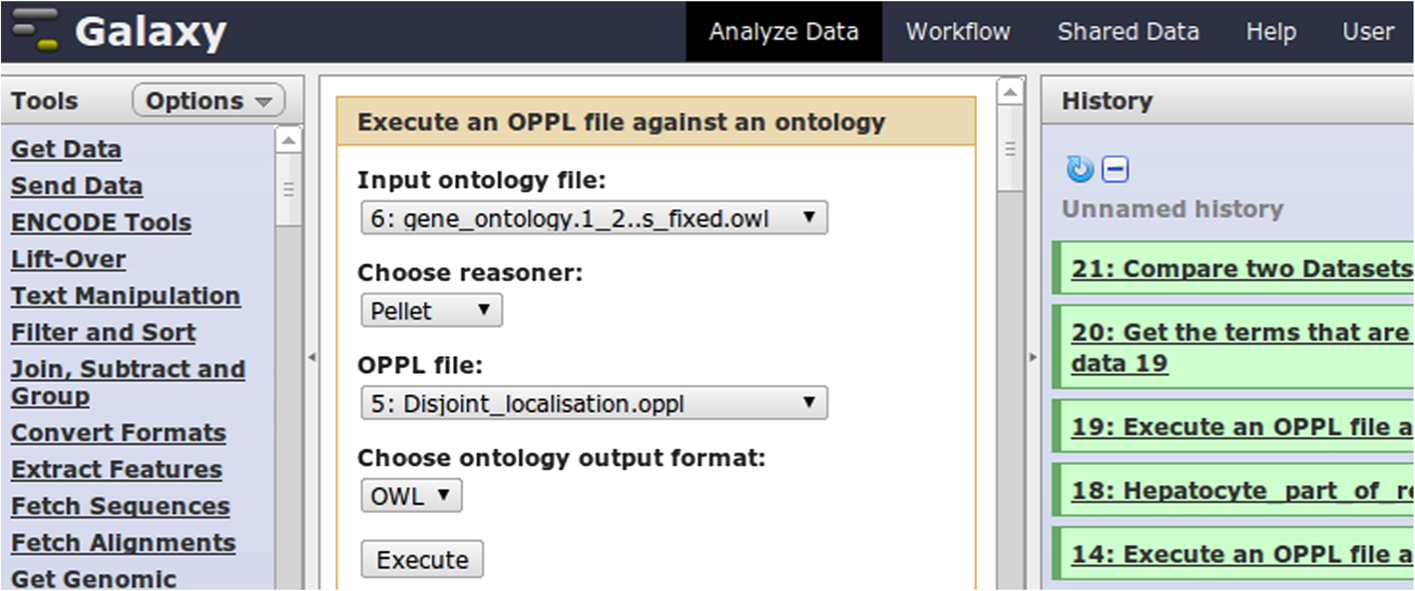
**OPPL-Galaxy architecture.** The inner circle represents the OPPL wrapper and the outer one Galaxy. Galaxy manages the data and parameters that will be passed to the OPPL wrapper. In order to pass, for instance, an ontology to the OPPL wrapper, the ontology must be first uploaded to Galaxy (or passed to it from the output of another Galaxy tool). Also, Galaxy manages the output of the OPPL wrapper: it can be redirected to other Galaxy tools or downloaded and saved as a standalone file. The OPPL wrapper coordinates the OPPL API (to parse the OPPL script and execute it), the OWL API (to read/write ontologies from stdin/to stdout and perform changes), and the chosen reasoner (to perform inferences).

OPPL can be executed through the graphical interface of Protégé [27] and Populous. Despite those possible means of manipulating ontologies, OPPL cannot be used as part of a workflow, limiting the possibilities of including other bioinformatics analysis tools, unless a tailored Java program is written using the OPPL API. OPPL-Galaxy fills that gap by offering an enhanced version of OPPL that can be used in combination with other Galaxy tools. To that end, an OPPL wrapper was developed as a mediator between Galaxy and both the OPPL 2 API [28] and the OWL API [29] (Figure 3).

**Figure 4:**
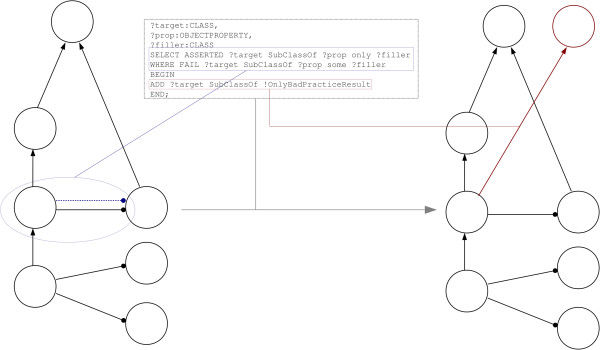
**OPPL-Galaxy Web interface.** The OPPL-Galaxy Web interface is displayed in the middle pane. In the left pane, a list of standard Galaxy tools is shown; in the right pane, a sample of a history of the executed tasks is shown.

OPPL-Galaxy takes as input a target ontology and an OPPL script: both artefacts are uploaded to Galaxy by the user or produced as output by another Galaxy tool. It generates a new ontology that has been changed according to the instructions defined in the OPPL script, thus axioms are added or removed. The OPPL-Galaxy Web interface presents the following options (Figure 4): 

• Target ontology: the input ontology that will be modified by the OPPL script. Since OPPL-Galaxy relies on the OWL API for loading and saving ontologies, it can load ontologies in the following formats: OBOF [30], OWL (RDF/XML, OWL/XML, Functional OWL Syntax, MOS), Turtle, and KRSS.

• OPPL script: a flat file containing the OPPL script that, when executed, will perform the desired changes in the target ontology. This file may be created by using the Protégé OPPL plugin *via* the OPPL text editor (with autocompletion), the OPPL script builder, or the OPPL macros tab (see the OPPL manual [31] for details on how to create OPPL scripts).

• Output format: the format of the output ontology, either OBOF or OWL (RDF/XML).

• Choose reasoner: the reasoner to be used for performing the inference, Pellet [32], HermiT [33], FaCT++ [34], or Elk [35].

The output ontology can be reused as input for other Galaxy tools like ONTO-toolkit [36], or downloaded from the Galaxy Web interface so that it can be used outside Galaxy, for example with Protégé or OBO-Edit [37].

OPPL-Galaxy includes various modules with diverse functionality, apart from executing OPPL scripts. Additionally, other tools are exploited as part of the use cases (NCBO-Galaxy [38], SPARQL-Galaxy, GO::TermFinder). See Table 1 for details.

**Table 1:** OPPL-Galaxy distribution and related Galaxy tools

	**OPPL-Galaxy bundle**
OPPL	Executes OPPL scripts
OWL Query	Perform DL (Description Logics) queries against OWL ontologies, returning a list of namedentities that satisfy the query
OPPL Query	Perform OPPL queries, thus, queries that mix MOS with variables
Inference	Add the inferred axioms to the input ontology as asserted axioms, generating a new ontologythat includes all the axioms
Merge	Resolves the import axioms and adds the imported ontology to the input ontology file
	**NCBO-Galaxy bundle**
The NCBO-Galaxy bundle includes modules for retrieving ontologies, extracting subtrees from ontologies,	
search for terms in ontologies, annotate texts against ontologies, *etc.* using NCBO Web services. See [38] for details	
	**SPARQL-Galaxy bundle**
SPARQL-Galaxy includes a tool for performing SPARQL queries on an OWL (RDF/XML) ontology;	
it can be downloaded from the Galaxy Tool Shed (http://toolshed.g2.bx.psu.edu), under ‘Ontology manipulation’.	
	**Galaxy-OBO**
Galaxy-OBO [39] is a fork of Galaxy that includes wrappers for common tools like GO::TermFinder [40]	

This table provides a detailed list of the OPPL-Galaxy tools and other tools that are executed in the workflows of the use cases.

## Results

This section provides use cases not only demonstrating the utility of OPPL-Galaxy but also showing, through examples, how to use it. The use cases are described in detail in [41]. All the use cases are provided as Galaxy workflows for users to be able to execute them without having to rebuild the use case from scratch. The URLs of the workflows are summarised at Table 2.

**Table 2:** Galaxy workflows for reproducing the use cases

**Name**	**Galaxy workflow**
Basic usage	http://biordf.org:8090/u/mikel-egana-aranguren/w/basic-usage-1
Ontology debugging andevaluation ^∗^	http://biordf.org:8090/u/mikel-egana-aranguren/w/ontology-debugging-and-evaluation
Complex querying of GO	http://biordf.org:8090/u/mikel-egana-aranguren/w/complex-querying-of-go
Expansion of gene productannotations through GOstructure	http://biordf.org:8090/u/mikel-egana-aranguren/w/expansion-of-gene-product-annotations-through-go-structure
Selective extraction of modulesfrom GO for term enrichment	http://biordf.org:8090/u/mikel-egana-aranguren/w/selective-extraction-of-modules-from-go-for-term-enrichment
OWL TBox to ABoxtransformation for assistingSPARQL queries	http://biordf.org:8090/u/mikel-egana-aranguren/w/owl-tbox-to-abox-transformation-for-assisting-sparql-queries

The name of the use case (as per section name) is provided in the left column; the URL of the Galaxy workflow is provided in the right column. In order to execute a workflow, the datasets (ontologies, OPPL scripts, GAFs, *etc.*) must be taken from the history (http://biordf.org:8090/u/mikel-egana-aranguren/h/oppl-galaxy-use-cases-for-jbs) or the workflow can be reproduced manually with the same datasets, by uploading them. The workflow “Ontology debugging and evaluation” obtains the ontologies directly from NCBO services. For detailed instructions, see http://wilkinsonlab.info/OPPL-Galaxy. All the workflows can be reproduced in a local Galaxy installation; in order to do so, the workflows and datasets can be downloaded from http://biordf.org:8080/JBSusecases.tar.gz.

### Basic usage

**Figure 5:**
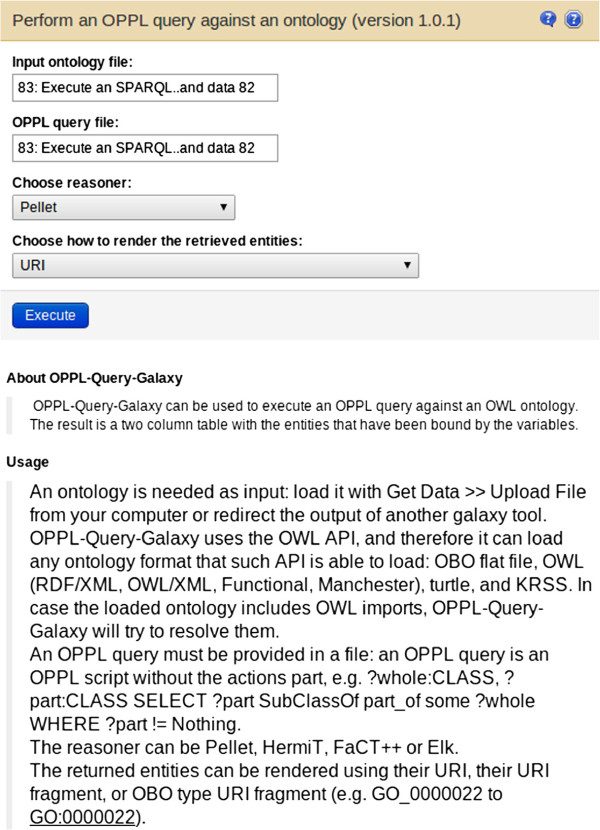
**Basic usage.** The OPPL engine takes the target ontology and OPPL script as inputs, and generates a new ontology changed according to the OPPL script. The OPPL script queries the reasoner for a class with a certain restriction (SELECT... WHERE clause, blue) and adds another restriction to the retrieved class (ADD clause, red).

The OPPL-Galaxy bundle includes a simple OPPL script for testing purposes that works with the test ontology also included in the bundle (Figure 5). The OPPL script is described as follows to help the reader understand the remainder of the use cases (more OPPL examples can be found at the OPPL scripts collection [42]):

Lines 1 and 2 show the declaration of two variables (?process and ?agent) and their type (CLASS). These variables represent (sets of) OWL classes. Then, line 3 introduces a SELECT clause, which is processed by OPPL and sent to the reasoner asking for the classes that are subclasses of the anonymous expression participates_in some ?process: the expression is written in MOS and it mixes named entities of the ontology (the property participates_in) with variables (?process and ?agent, representing sets of classes). Later, in line 4, the classes retrieved as members of the variable ?agent are checked for satisfiability (?agent != Nothing). Finally, the axiom SubClassOf participates_in only ?process is added (ADD) to the input ontology, resolving ?agent and ?process to all the classes that have been bound and combinations thereof.

### Ontology debugging and evaluation

Ontology debugging (the process of fixing defects in an ontology) can be a daunting activity, especially when the ontology the scientist is working with has not been developed in-house and/or if it presents a complex axiomatisation over many entities. OPPL-Galaxy can be used for detecting and fixing certain structures that are considered bad practice (antipatterns) or at least ‘suspicious’. The detection of antipatterns also offers a ‘picture’ of the ontology: it can be used to evaluate the overall structure of the ontology as one of the criteria to judge its quality. OPPL-Galaxy provides a means of defining antipatterns as ‘test units’ that can be run automatically against a set of ontologies, as part of Galaxy workflows.

The notion of antipatterns in ontologies has already been introduced [43,44]. For example, [44] mentions using the OWL universal restriction (only) without any other restriction on the same property (*e.g.*some) as a potential antipattern (exclusive universal). This is due to the fact that, the only restriction, on its own, can be trivially satisfied by an unsatisfiable (empty) class, *e.g.*A subclassof p only (B and C) can be satisfiable even when B disjointWith C, since the semantics of only state that *if* there is a relation, it must be to (B and C), or *none*: (B and C) is empty and therefore would satisfy the *none* case.

**Figure 6:**
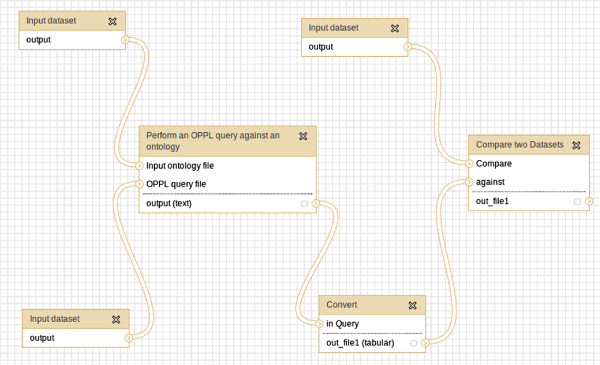
**Ontology debugging and evaluation.** This script detects any class that has a universal restriction *without* an existential restriction (dotted blue line). It adds a subClassOf OnlyBadPracticeResult axiom (red arrow) to any matching class.

The exclusive universal structure can be easily detected in, for example, BioPAX [45], by the following OPPL script (Figure 6):

This script detects the exclusive universal structure^b^and adds all the classes that present it as subclasses of OnlyBadPracticeResult, a class created on the fly if it does not exist in the ontology (! symbol). Note the use of the ASSERTED keyword (only the asserted axioms, not the inferred ones, are taken into account: the reasoner is deactivated for querying in order to improve performance) and the FAIL keyword (negation as failure, which is out of OWL semantics, is used to detect *absent* existential restrictions).

**Figure 7:**
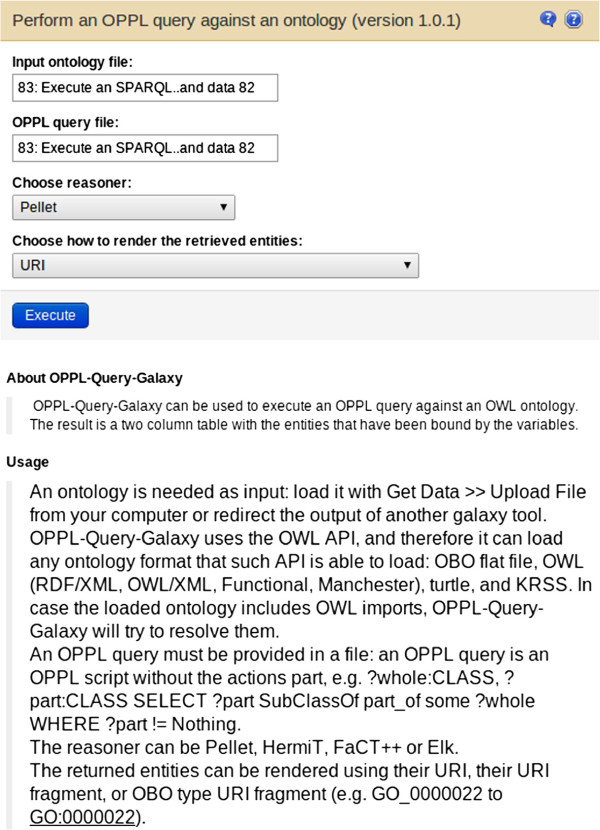
**OPPL query tool.** Web interface of the OPPL query tool.

The ontology can also be simply queried, without modifying it, by using the OPPL-Query tool (See Table 1 and Figure 7):

The exclusive universal structure can also be modified by adding an existential restriction to every universal restriction:

Even though the exclusive universal structure might be considered as a legitimate modelling decision, it is recommendable, to make sure there is no trivially satisfiable classes, to add existential restrictions on the fly (and possibly to make entities disjoint), apply reasoning to detect trivially satisfiable classes, and then remove the existential restrictions again. Such procedure can be automatically performed using OPPL-Galaxy. An alternative would be to check the consistency of the filler, *e.g.*?filler subClassOf owl:Nothing, with the reasoner activated, instead of checking for the exclusive universal structure [46].

More antipatterns can be found in the collection presented in [43]: 

• Logical Antipatterns (LAP): modelling errors that are detectable by an automated reasoner, *e.g.* unsatisfiable classes.

• Non-Logical Antipatterns (NLAP): modelling errors that are not detectable using a reasoner, usually created by the developer due to a misunderstanding of the language semantics (the logical consequences of the axioms stated in the ontology).

• Guidelines (G): alternative, simpler axiomatic expressions of the same knowledge.

Synonym Of Equivalence (SOE) is an example of a NLAP. Such type of antipattern describes the situation in which two classes are declared as being equivalent and both pertain to the same ontology (*i.e.*, they have not been imported). Generally, that means that the developer intends to model a synonym, which should be an rdfs:label string, as a whole class. Such structure can be easily detected, for example, in the NIF Gross Anatomy ontology [47], using the following script (which also removes the non-desired structure):

We do not claim that these structures (exclusive universal in BioPAX and SOE in NIF Gross Anatomy) are erroneous *per se*. We rather state that, according to the experience of the authors of [43,44], and ours, they are modelling practices that may yield unexpected results when automated reasoning is applied downstream. Therefore, a scientist who might reuse those ontologies should be aware of the existence of the mentioned antipatterns.

OPPL-Galaxy is a straightforward, powerful and flexible tool to detect antipatterns *en masse* when executed as a Galaxy workflow: a scientist can have a collection of antipatterns of her choice ready to be applied in any ontology she wants to reuse (any antipattern can be defined by her, since OPPL is, roughly, a superset of OWL). The full process can be automated, defining once what ontologies to obtain and then adding antipatterns to the collection as needed. Once the workflow has been executed and the antipatterns detected in the target ontology, she can decide if the ontology meets her requirements. Additionally, OPPL-Galaxy can be used to modify the ontologies that do not meet her requirements, within the same workflow.

### Complex querying of GO

**Figure 8:**
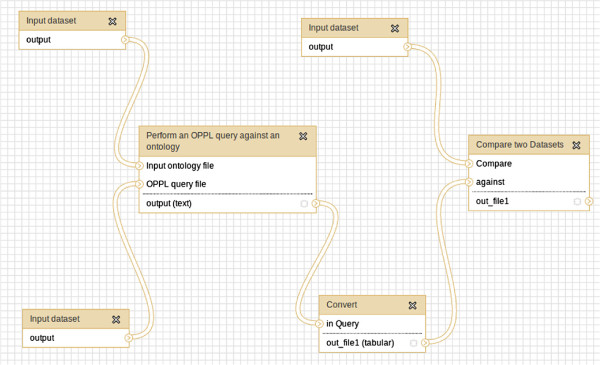
**Complex querying of GO (as shown in Galaxy).** OPPL-query workflow for quering GO against GAFs. The result is a list of proteins of interest.

**Figure 9:**
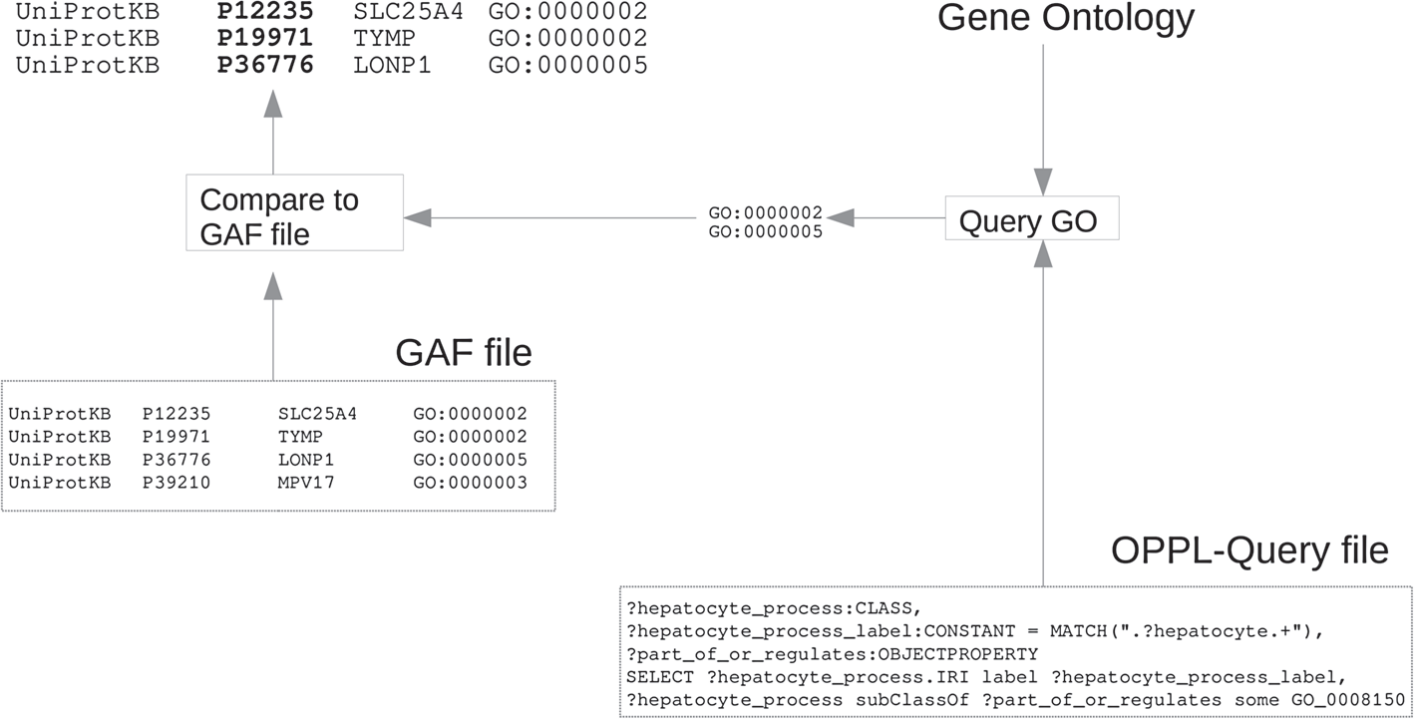
**Complex querying of GO (details).** Detailed depiction of the workflow shown in Figure 8.

OPPL-Galaxy can be combined with other Galaxy-enabled tools to build advanced workflows such as the one shown in Figures 8 and 9. This workflow can be used by a scientist to pose a complex question against GO, namely ‘What are the proteins that act on processes that involve hepatocytes and are part of or regulate other biological processes?’. Posing such a complex question requires different steps that can be performed with OPPL and stored for further analysis with the help of Galaxy.

The workflow executes the OPPL query tool and the Galaxy tool for comparing two data sets (included in the standard Galaxy distribution, in ‘Join, subtract and group’). Thus, this workflow combines Galaxy tools to retrieve exactly the proteins that the scientist defined in her plain-english query, which is translated into a machine interpretable form, as discussed below.

The OPPL script queries GO for the terms that have ‘Hepatocyte’ as part of their names and that are related, *via*part_of or regulates, to a biological process:

Then, the Galaxy tool for comparing two data sets is used to extract the proteins involved in the resulting processes of interest, using the GO terms as keys against a Gene Association File (GAF) [48]. The result of this comparison is a list of the protein identified as of interest.

This workflow demonstrates some of the main advantages provided by OPPL-Galaxy: on one hand, this type of analysis can only be performed, effectively, with OPPL (see below). On the other hand, the unique capabilities of OPPL are enhanced due to the fact that they are executed within Galaxy: the process can be repeated with any new version of GO or GAFs, it can be shared with other scientists, combined with other tools, and modified or ran in parallel with minimum effort.

OPPL enables a unique set of capabilities for analysing ontologies. It can mix, for instance, text manipulation (in this case the regular expression (".?hepatocyte.+")) and automated reasoning (in this case subPropertyOf axioms, and subClassOf and part_of transitivity) as part of the same query. It also enables the ability to refer to groups of entities *via* variables, a feature which is outside the standard OWL semantics, unless explicit axioms are codified into the ontology (*e.g.* equivalent property axioms): part_of and regulates are represented by the same variable ?part_of_or_regulates, including the subproperties negatively_regulates and positively_regulates, due to the OWL semantics (subPropertyOf).

### Expansion of gene product annotations through GO structure

**Figure 10:**
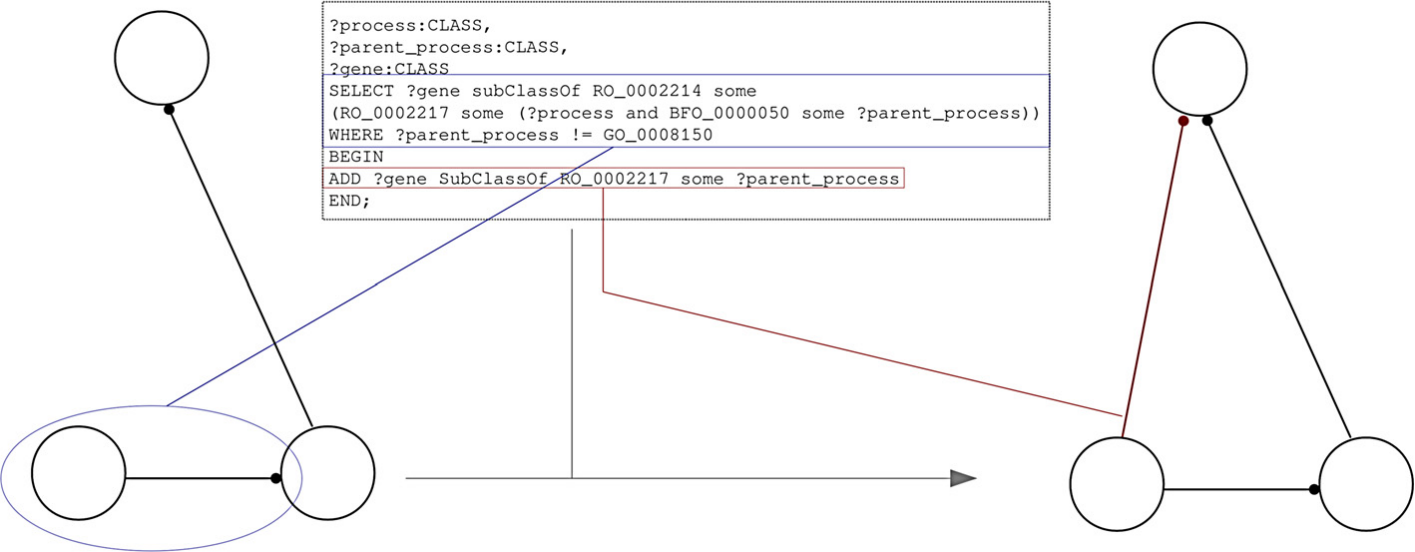
**Expansion of gene product annotations through GO structure.** This workllow starts from an OWL ontology that includes GAF information, produced by OORT. The script detects the structure ?gene subClassOf RO_0002214 some (RO_0002217 some (?process and BFO_0000050 some ?parent_process)) (Simplified depiction) and adds a new restriction to every matching class.

GO annotations are provided independently of the ontology itself, in GAFs. However, being able to access gene products linked to GO through annotations is a useful feature for queries and other analyses [49]. One of the tools that can be used to merge GAFs with GO is OORT (OBO Ontology Release Tool) [50]: it offers, for a given ontology version, the possibility of checking its syntactic and semantic quality, before releasing it. It also includes the functionality to transform GAFs into ontologies, in doing so linking, in the same ontology, gene products with their GO terms. This gives the possibility of directly exploiting the structure of GO against the gene product data: For example, if gene product G is capable of function F and F is part of P (as per GO structure), then G is also capable of G. Such semantic expansion of gene product information can be performed using OPPL-Galaxy, providing an ontology generated by OORT that includes the link between gene products and their GO terms as input. For example, the relations of the gene product Atu0514 (subClassOf has_prototype some (actively participates_in some 'chemotaxis on or near host involved in symbiotic interaction')) can be expanded with the following script (this use case was obtained from [51], see Figure 10):

This script queries the ontology and expands any gene product - GO term relation according to the partonomy hierarchy. As a result, the new axioms for Atu0514 read as follows:

This new ontology can be used for further analyses.

### Selective extraction of modules from GO for term enrichment

A typical use for GO is to perform an over-representation analysis of genes expressed in micro-array experiments, also known as enrichment analysis. To that end, a module or subset from GO is usually extracted, as recommended in [36], so that the statistical values of the analysis could be sounder (*i.e.*, the bias that might be introduced by considering other modules is diminished since the gene product space is smaller).

**Figure 11:**
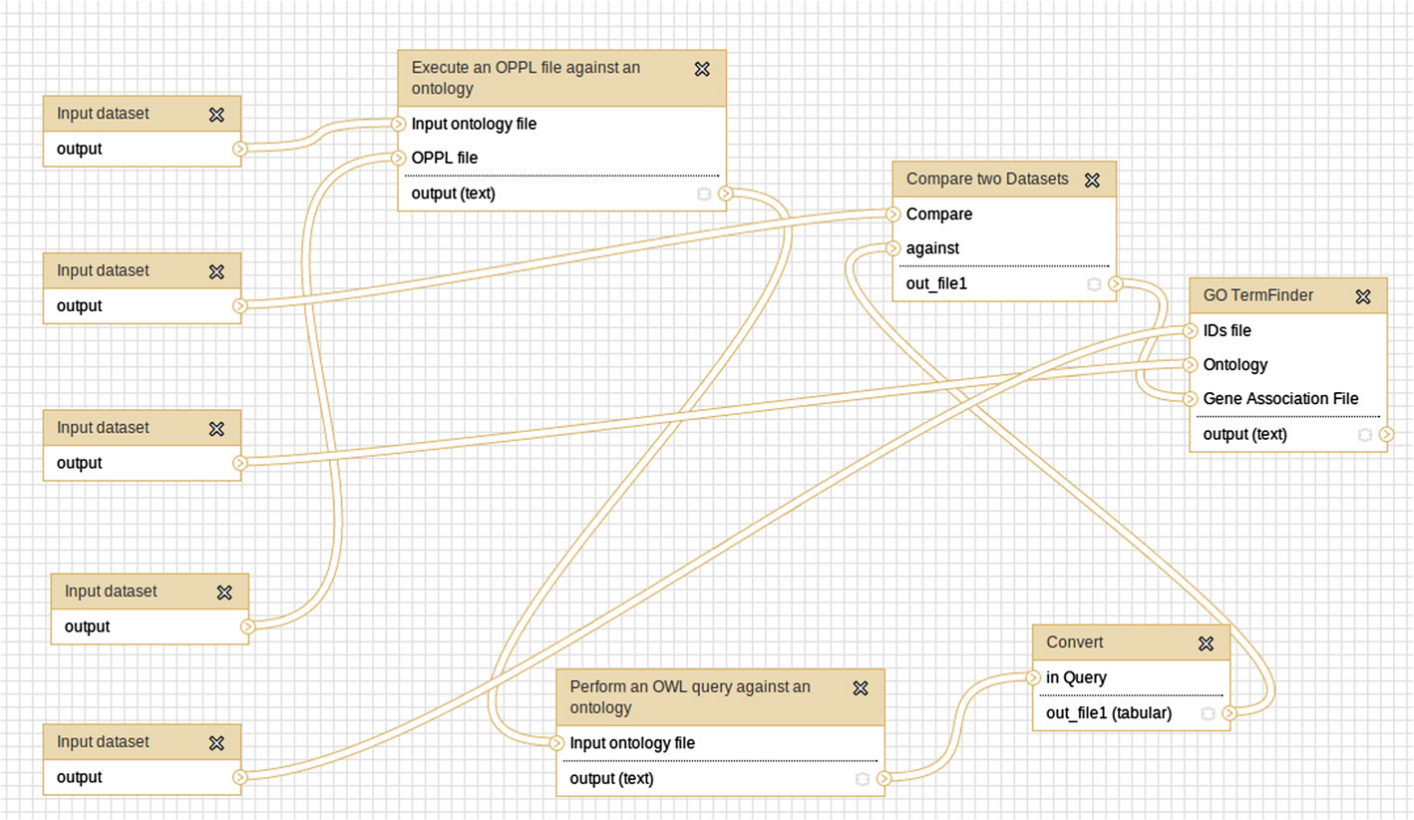
**Selective extraction of modules from GO for term enrichment (as shown in Galaxy).** In this workflow a reduced GAF is obtained by querying GO (*i.e.*, extracting a module) and comparing the retrieved GO terms with the GO terms from the GAF. The resulting reduced GAF is used to perform an enrichment analysis with GO::TermFinder.

**Figure 12:**
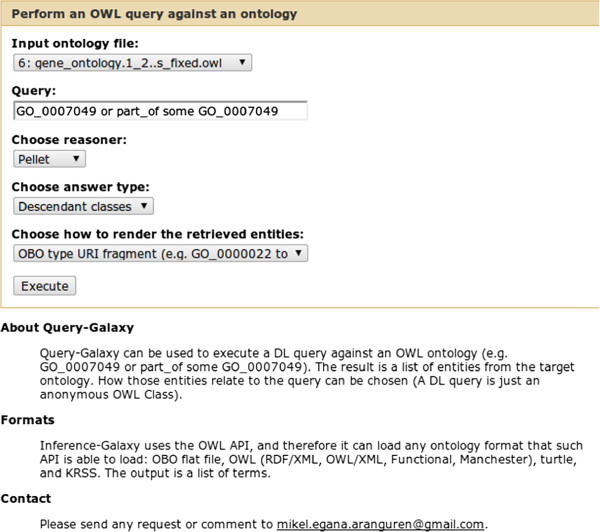
**OWL query tool.** Web interface of the OWL query tool.

OPPL-Galaxy can be combined with OWL-Query-Galaxy to extract a module (Figure 11). The extent of such module can specified with OPPL-Galaxy, for example by adding transitivity to the regulates object property (as a result the module holds more terms):

The resulting ontology can be later queried with the OWL-Query-Galaxy tool (also part of OPPL-Galaxy, see Figure 12), to obtain the module, *i.e.* a list of GO terms, that can be then used to perform the enrichment analysis by using other Galaxy tools like GO::TermFinder:

OPPL performs, in this case, the same function as ONTO-toolkit but in a more flexible way. Another advantage of this procedure is that it can be executed every time GO is updated, *i.e.*, scientists can easily extract different modules with a few clicks, and compare them using Galaxy tools.

### OWL TBox to ABox transformation for assisting SPARQL queries

Making SPARQL queries against TBox axioms of an RDF/XML OWL ontology is awkward. OWL punning (see bellow) can be used to add an instance to every class and be able to do succinct SPARQL queries while retaining the original TBox semantics [52] (However, the resulting ontology has new semantics due to the addition of ABox assertions).

OWL punning is a feature provided by OWL 2 that makes it possible for different entities to share the same URI [53]. The ‘punned’ entities that share the same URI are differentiated by the reasoner using their axiomatic context. Punning can only be used within precisely defined limits: for instance, the same URI cannot be shared by both a class and a data type property.

**Figure 13:**
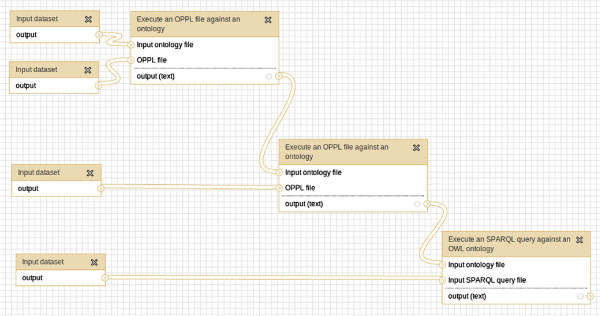
**OWL TBox to ABox transformation for assisting SPARQL queries (as shown in Galaxy).** In this workflow two OPPL scripts are used: the first one adds an instance to every class with the same URI and the second one adds an RDF triple for every existential restriction.

**Figure 14:**
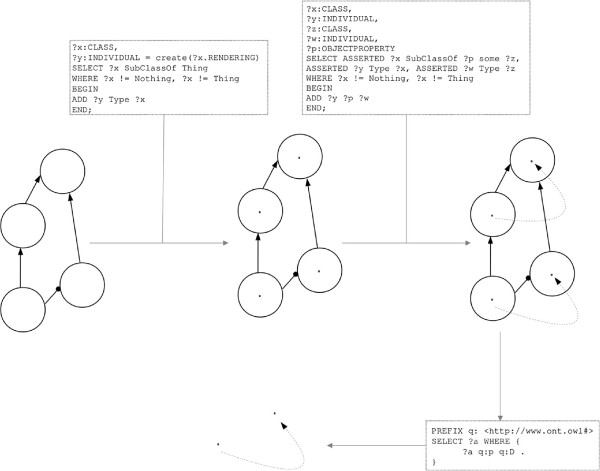
**OWL TBox to ABox transformation for assisting SPARQL queries (details).** Detailed depiction of the workflow shown in Figure 13.

Therefore, to have both classes (for DL or OWL syntactic queries) and individuals (for more ‘comfortable’ SPARQL queries), it makes sense to add, for every class, an individual with the same URI, *i.e.* to use OWL punning in the ontology. The following OPPL script can be used for such a task (Figures 13 and 14):

By applying this simple script a ‘punned’ ontology can be quickly obtained: the script adds an individual as a member of each class, with the same URI as the class, except in the case of owl:Thing and owl:Nothing (line 4). It is worthy noting that the RENDERING keyword in OPPL refers to the rendering method used in Protégé 4 for entities: URI fragment, rdfs:label, QName, *etc.* (OPPL-Galaxy uses the default, URI fragment). As a result, an ontology in which each class has an individual with the same URI is obtained. An RDF triple for every existential restriction can be added to the punned ontology by executing the following script (using the punned ontology as input):

This script will only work for existential restrictions, *i.e.* it will not transform universal restrictions to triples^c^. Therefore, it will completely transform an ontology that only presents existential restrictions, like GO. By using such scripts sequentially in a Galaxy workflow, a ready-to-use (OWL) RDF representation can be obtained to be submitted to a Galaxy tool for executing SPARQL queries (Table 1).

## Discussion

One of the most important applications of OPPL is the axiomatic expansion of an existing ontology. The definition of complex modelling made by an ontologist is expanded, through the script execution, to different parts of the ontology itself, saving in this way time and effort. Such complex modelling can be stored in a script, which can be reused at any time in order to (re)apply precisely defined ontology patterns. Thus, OPPL abstracts away the repetitive task of implementing common axiom patterns found in ontologies and parameterising them with concrete entities. Using OPPL when building ontologies ensures the repeatability and style consistency of the modelling since such modelling is performed by executing a script. Moreover, OPPL allows experimentation with modelling choices: design options can be stored in a script and by simply executing such script and inspecting the results, the ontologist can rapidly try out complex modelling and revise decisions as necessary.

OPPL provides a simple, flexible and expressive language for maintaining ontologies as well as for keeping track of the changes themselves. By using OPPL, in contrast to a direct OWL API implementation, users profit from less complex scripting that does not require the overhead of a Java program, yet retains the complexity and capabilities needed to work with OWL ontologies in a fully expressive manner. OPPL scripting is not a simple task; nonetheless, OPPL scripts do afford a unique programmatic way to manipulate OWL ontologies in a pattern based manner that avoids many of the issues with manual crafting of individual axioms.

The only tool that offers a functionality similar to OPPL is Thea [54]. Thea, however, requires the ontologist to be able to program her axioms in Prolog. OPPL, in contrast, requires a knowledge of its scripting syntax, which is an extension of MOS (which in turn is an OWL syntax designed for human use and readability) based on an intuitive set of keywords (such as ADD, REMOVE, *etc.*). Therefore, the OPPL syntax learning curve is not that steep for an ontologist who is familiar with the OWL syntax. On the other hand, Galaxy enhances the mentioned features of OPPL by embedding them in an infrastructure that provides persistence, shareability and reproducibility of analyses, combination with other tools, *etc.* To the best of our knowledge, there is no other Galaxy tool comparable to OPPL-Galaxy, except ONTO-toolkit. However, ONTO-toolkit offers different, complementary functionalities to the ones offered by OPPL-Galaxy and as a matter of fact they can be combined to obtain meaningful results.

OPPL-Galaxy is a seminal prototype that is regularly improved. The following list collects a set of prospective features: 

• Loading ‘local’ imported ontologies by uploading them to Galaxy (Currently only remote URIs are resolved).

• Load ontologies by their URI.

• Configurable querying and rendering (URI fragment, rdfs:label, QName, *etc.*).

• Standalone OPPL assertions processing (*e.g.*ADD phagocyte subClassOf cell).

• Support for OWLlink [55] and RACER [56] reasoners.

• Other output formats apart from RDF/XML.

• In the case of the inference module, support for more inferences like data property assertions, different individuals assertions, *etc.*

• A tool for wrapping the ontology modularisation function of the OWL API.

Performance might be an issue while working with OPPL-Galaxy [18], since automated reasoning on especially large, complex biomedical ontologies is usually resource demanding [57], even considering that OPPL-Galaxy will normally work in a server with considerable memory. As performance typically depends on the implementation of the automated reasoners, it is expected to improve in the future, since reasoners are becoming more efficient. Also, Galaxy can used in a cloud computing setting such as Amazon EC2 [58].

## Conclusions

The success of the application of the Semantic Web technologies in Life Sciences not only relies on building ontologies and fine-tuning or setting standards, but also on augmenting the scientists’ toolbox with tools that can be easily plugged into frequently-used data analysis environments such as Galaxy. Galaxy facilitates the combination of several bioinformatics tools within a single Web interface. Since OPPL-Galaxy can be used as part of the Galaxy framework as an ontology manipulation tool, it can be exploited in combination with other Galaxy tools. That is, precisely, what sets OPPL-Galaxy apart from other ontology tools that offer similar functionality: it can be used with the actual data and tools that life scientists use on a daily-basis, rather than in isolation. By embedding tools like OPPL in genomic science frameworks like Galaxy, the user awareness of such type of application of the semantic technologies in Life Sciences could increase, thus enabling more sophisticated analyses of biomedical information.

The OPPL syntax extends that of OWL with a set of intuitive keywords; therefore, the learning curve of any user minimally fluent in OWL should be relatively shallow. This means that OPPL-Galaxy provides a powerful and (indirectly) familiar tool for automating ontology curation processes that otherwise would need considerable human resources and/or might produce incomplete or erroneous results. The OPPL scripts described in the results section are relatively simple, yet they show how users could benefit from this tool to enhance their ontology development and exploitation tasks, like debugging, rewriting and performing axiomatic enrichment *via* ODPs. Specially in the case of ODPs, a well-known ontology engineering practice, OPPL-Galaxy offers the ideal setting for their application, since such ODPs can be shared as ready-to-execute Galaxy workflows, saving time and effort. More complex OPPL scripts would undoubtedly yield even greater benefits, particularly if combined in workflows (*e.g.* debugging and rewriting sequentially and sending the output to other Galaxy tools).

Examples of Galaxy workflows that combine different OPPL scripts with other Galaxy tools are provided in the use cases ‘Complex querying of GO’, ‘Selective extraction of modules from GO for term enrichment’, and ‘OWL TBox to ABox transformation for assisting SPARQL queries’. Other sophisticated analyses can be performed with workflows exploiting OPPL-Galaxy, like more fine-grained axiomatic enrichment of biomedical ontologies [18,59-61]. The diversity and functionality of Galaxy workflows involving OPPL-Galaxy depend only on the user.

In summary, OPPL-Galaxy offers the possibility of automating ontology manipulations in a reproducible, versatile, persistent and shareable fashion, within a context in which the result of such manipulations can be sent directly to other tools in order to further build or enhance analysis workflows. Therefore, OPPL-Galaxy should, on the one hand, be of interest for the life scientists that exploit ontologies to analyse biomedical information, and, on the other hand, for bio-ontologists that continuously maintain ontologies and are concerned by their quality.

## Endnotes

^a^Strictly following this convention would result in restrictions being represented as lines going out of dotted circles (A condition in an OWL class is the anonymous class formed by the individuals that have the relation). However restrictions have been simplified, omitting the anonymous class, for the sake of clarity.^b^This script detects any case in which a universal restriction is used in the absence of an existential restriction. Therefore, it would (wrongly) flag as an instance of the antipattern, for example, a universal restriction and an exactly restriction used together. A more thorough script is feasible but out of the scope of this paper.^c^The reason for not including universal restrictions is that, in the case of GO, only existential restrictions are present in the ontology; nothing prevents the user from adding a further statement so as to also capture universal restrictions, but in the case of GO no entities would be retrieved.

## Availability and requirements

• **Project name:** OPPL-Galaxy.

• **Project home page:**http://wilkinsonlab.info/OPPL-Galaxy. We provide a public instance of Galaxy with OPPL-Galaxy installed on it, including Galaxy tools related to the use cases (ONTO-toolkit, NCBO-Galaxy, Annotation, SPARQL-Galaxy): http://biordf.org:8090. The Galaxy bundle for local installation can be downloaded at http://toolshed.g2.bx.psu.edu/, under the category ‘Ontology manipulation’. The bundle includes the software itself (along with the necessary third-party libraries and XML tool files), sample scripts and ontologies, and instructions on installation and usage.

• **Operating system(s):** it is recommended that OPPL-Galaxy be deployed in a UNIX-based machine (GNU/Linux, Mac OS X, BSD, *etc.*) since it uses standard UNIX redirection (MS Windows ^TM^ is not officially supported by Galaxy).

• **Programming language:** Java and Python.

• **Other requirements:** a working Galaxy installation is needed (http://galaxy.psu.edu/).

• **License:** General Public License (http://www.gnu.org/copyleft/gpl.html). Source available at the Galaxy tool shed mercurial repository (http://toolshed.g2.bx.psu.edu/repos/mikel-egana-aranguren/oppl).

## Abbreviations

DL: Description Logics; BioPAX: Biological Pathway Exchange; GAF: Gene Association File; GO: Gene Ontology; KB: Knowledge Base; MOS: Manchester OWL Syntax; NCBO: National Center for Biomedical Ontology; NLAP: Non-Logical Antipattern; OBO: Open Biomedical Ontologies; ODP: Ontology Design Pattern; OORT: OBO Ontology Release Tool; OPPL: Ontology Pre Processor Language; OWL: Web Ontology Language; RACER: Renamed ABox and Concept Expression Reasoner; RDF: Resource Description Framework; SOE: Synonym Of Equivalence; SPARQL: SPARQL Protocol and RDF Query Language; URI: Uniform Resource Identifier; W3C: World Wide Web Consortium.

## Competing interests

The authors declare that they have no competing interests.

## Authors’ contributions

MEA developed OPPL-Galaxy, designed some use cases, and contributed to the text. JTFB and EA tested the use cases, created the Web for them and contributed to the text. CM contributed to the use cases and to the text. ARG developed SPARQL-Galaxy. MDW revised the manuscript and supervises the Biological Informatics Group and funding. All authors read and approved the final manuscript.
